# Patterns of Inflammation in Experimental Autoimmune Uveitis and Their Correlation to Optical Coherence Tomography Findings in Human Uveitis

**DOI:** 10.3390/ijms27041618

**Published:** 2026-02-07

**Authors:** Benedikt Schworm, Tarek Ghannoum, Stephan Thurau, Gerhild Wildner

**Affiliations:** 1 Department of Ophthalmology, LMU University Hospital, LMU Munich, Mathildenstr. 8, 80336 Munich, Germany; t.ghannoum@med.uni-muenchen.de; 2 Section of Immunobiology, Department of Ophthalmology, LMU University Hospital, LMU Munich, Mathildenstr. 8, 80336 Munich, Germany; stephan.thurau@med.uni-muenchen.de

**Keywords:** uveitis, chorioretinitis, vasculitis, immunohistochemistry, molecular pathomechanisms, immunology, animal model, OCT imaging

## Abstract

Experimental autoimmune uveitis (EAU) in rats is a pivotal model for understanding the immunological mechanisms of human uveitis and developing therapies. In humans, optical coherence tomography (OCT) allows for the in vivo detection of characteristic findings in active uveitis, as well as sequelae of inflammation. The objective of this study was to correlate OCT findings in patients with uveitis with retinal histologies from two rat models of EAU caused by T cells with different autoantigen specificities and well-known underlying immunological pathomechanisms. Patients with various noninfectious uveitis subtypes underwent imaging using an ultra-widefield swept source or conventional OCT. Histological cryosections from rat eyes with experimental autoimmune uveitis were stained for T cell and/or macrophage markers. Typical human OCT findings were reproduced in the experimental animal model. Hyperreflective signals observed on OCT corresponded to lymphocyte infiltration in histological sections. This infiltration was typically found as vasculitis in the perivascular regions and snowballs in the posterior hyaloid. There was lymphocyte and macrophage infiltration of the retina through the retinal vessels and the retinal pigment epithelium. Comparing in vivo OCT imaging of human uveitis with corresponding histologies from rat models improves our understanding of the type of inflammation, extent of tissue destruction, and immunopathogenesis.

## 1. Introduction

Uveitis is the collective term for inflammatory diseases of the intraocular structures. Based on the primary site of inflammation, it is classified as anterior, intermediate, or posterior [1]. This condition can range from mild to severe and can threaten vision. Patients with autoimmune uveitis often require continuous immunosuppressive therapy and regular monitoring of disease activity through ophthalmological assessments, such as slit-lamp examinations and indirect fundoscopies.

Over the past two decades, modern imaging techniques have become an important part of evaluating patients with uveitis. Among these modalities, optical coherence tomography (OCT) of the posterior pole has proven particularly valuable for visualizing retinal structural changes. Recently, OCT was incorporated into the minimum imaging requirements for various forms of non-infectious posterior uveitis [2]. Currently, its application mainly focuses on the posterior pole of the eye, particularly the macular region and the optic nerve head (papilla).

However, ultra-widefield imaging devices can now visualize peripheral retinal structures. These structures have been poorly characterized thus far in uveitis. Many imaging findings appear to correlate with active inflammation, while others indicate chronic disease, structural damage, and loss of retinal tissue. Since uveitis is an inflammatory disorder, it is likely that several of these imaging features result from immune cell accumulation and/or migration. Direct histological verification in humans is not feasible, because ocular biopsies pose a risk of vision loss.

Despite the increased resolution of OCT scans in recent years, many details remain hidden and we only have a general idea of the extent of retinal infiltration and destruction. Furthermore, we cannot identify the type of infiltrating cells. Therefore, we examined histological sections of rat eyes with experimental autoimmune uveitis (EAU) to better understand the types of cells and sites of retinal invasion. Correlation with the autoantigen specificities of the experimental models allows us to speculate about the expression, degradation, and presentation of the respective autoantigens. The gene expression profiles of the autoreactive T cells in rat models explain certain sequelae such as choroidal neovascularization and relapses [3,4,5,6,7].

Experimentally induced autoimmune uveitis (EAU) in animals exhibits disease patterns that closely resemble those observed in humans. EAU in Lewis rats has been thoroughly investigated in terms of histological images of retinal infiltration and destruction, as well as immune mechanisms. The two major autoantigens used to induce EAU in Lewis rats are the retinal S-antigen (S-Ag, arrestin) and the interphotoreceptor retinoid-binding protein (IRBP), along with peptides representing their most pathogenic epitopes [7].

The ocular autoantigens responsible for human uveitis remain unknown. Although antibody and T cell responses to S-antigen, IRBP, and some peptides have been demonstrated, the full spectrum of ocular autoimmune responses remains unclear [8,9]. Nevertheless, EAU in rats is a valuable model for human autoimmune uveitis. It has been used to detect pathomechanisms and test new potential therapies for uveitis. Finally, we have found striking similarities between retinal optical coherence tomography (OCT) images of patients with uveitis and histological retinal images of rats with EAU [10,11,12,13,14].

The S-Ag peptide PDSAg induces a chronic, clinically monophasic uveitis with choroidal neovascularization formation due to VEGF production by autoreactive T cells [6]. In contrast, immunization of rats with the IRBP peptide R14 results in a disease that spontaneously relapses, driven by the higher IFN-γ production of R14-specific T cells [3,4,6,13]. In S-Ag- and PDSAg-induced uveitis, we found that T cells and inflammatory cells are primarily recruited from the choroid and then migrate through the RPE. This suggests that the RPE or other antigen-presenting cells, such as choroidal macrophages, present the autoantigen. In contrast, IRBP/R14-induced EAU shows infiltration via retinal capillaries, which usually leaves the RPE intact [15]. The antigen specificities of the autoimmune responses and the types of lymphocytes and leukocytes that drive inflammation in human uveitis remain unknown.

Not only do autoreactive T cells recognize different retinal autoantigen epitopes, but they also differ in their secretion of cytokines and chemokines, as well as in their expression of various surface receptors. This has been demonstrated in rat models with autoreactive T cells that recognize peptides from S-Ag (PDSAg) and IRBP (R14) [4,5,13]. Depending on their cytokine profile, these cells can recruit different inflammatory cells, such as monocytes/macrophages or granulocytes. This results in either granulomatous/nodular (macrophages) or non-granulomatous (neutrophil granulocytes) ocular inflammation. In rat EAU, the predominant infiltrating leukocyte populations are monocytes/macrophages, though granulocytes are also present. Therefore, it is not possible to distinguish between the two types of uveitis in the rat model.

Nevertheless, we discovered significant similarities between human and experimental uveitis by comparing human optical coherence tomography (OCT) scans of various forms of posterior uveitis with sections of rat eyes with experimental autoimmune uveitis (EAU) and immunohistochemical staining. The aim of this study was to better understand the fine structure and extent of inflammatory signs and tissue destruction in the retina and vitreous in human uveitis, as well as the characteristics of invading cells.

## 2. Results

### 2.1. Different Sites of Ocular Infiltration by Inflammatory Cells

In rats, S-Ag-peptide-induced experimental autoimmune uveitis (EAU) typically involves retinal cell infiltration via the choroid and retinal pigment epithelium (RPE), resulting in RPE and adjacent retinal layer destruction. Additionally, we observed new vessel growth from the choroid, which was similar to what is seen in some patients with choroiditis. In contrast, R14-induced uveitis shows cellular infiltration primarily via the retinal vessels (in the outer and inner plexiform layers). This results in destruction of the retinal architecture while leaving the RPE largely intact [15]. This inflammatory pattern resembles human intermediate uveitis regarding vitreous infiltrates and peripheral periphlebitis.

Recently, we demonstrated a reactivation of previously inflamed retinal foci in the rat model, wherein GFP+ T cells preferentially invaded sites of prior tissue destruction, causing relapses [7].

**Figure 1:**
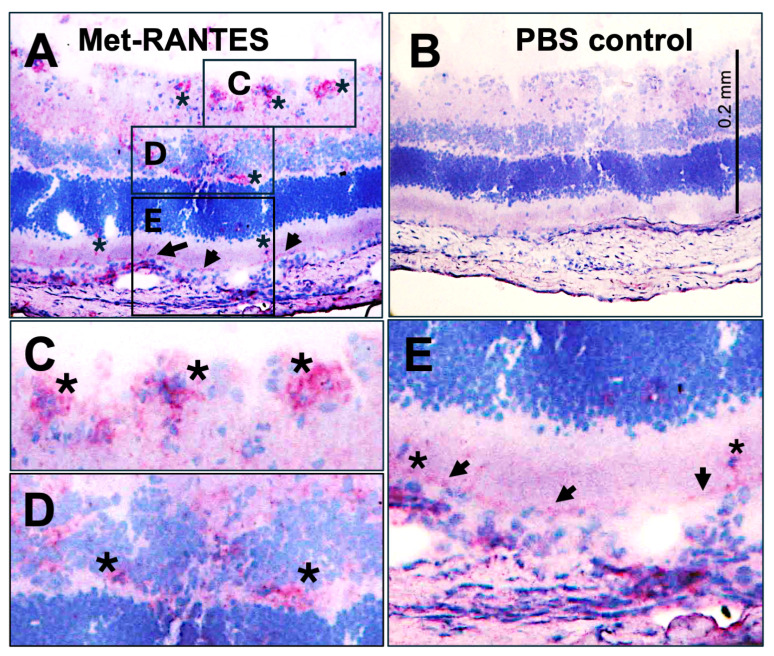
(**A**): Rat eye with mild R14-induced EAU stained for binding of Met-RANTES (red staining), visualized with anti-human RANTES antibody VL2 (asterisks and arrow). (**B**): PBS was added instead of Met-RANTES. Endogenous rat RANTES was not detected with VL2. (**A**) Met-RANTES bound to glycosaminoglycans and chemokine receptors CCR1 or CCR5 on RPE (arrows) and infiltrating cells ((**A**), asterisks, red staining). Zooms show (**C**) accumulation of infiltrating immune cells and potentially the blood vessels they had evaded from (asterisks); (**D**) immune cells (asterisks) infiltrating from capillaries in the outer plexiform layer; and (**E**) the outer nuclear layer, photoreceptors, RPE, and choroid (from up to down). Arrows mark the RPE, and asterisks mark single infiltrating immune cells.

The infiltration routes of immune cells differ between the two types of rat EAU. PDSAg-specific T cells and their recruited inflammatory cells preferentially evade the choroid. These cells infiltrate the retina by passing through the RPE, which forms the outer blood–retina barrier. In contrast, R14-specific T cells invade the eyes via the retinal vessels (e.g., in the outer plexiform layer) [15]. Staining rat retina sections with the chemokine receptor antagonist Met-RANTES revealed its binding to some infiltrating cells (Figure 1A,C,E, asterisks) and to the basolateral site of the RPE (Figure 1A,E, arrows). Met-RANTES strongly binds to glycosaminoglycans (GAGs) such as heparin and heparan sulfate, as well as to the chemokine receptors CCR5 and CCR1. These receptors are expressed in the choroid and RPE as well as on monocytes/macrophages and T cells in humans and rats. Although GAG binding of chemokines contributes to chemoattraction, signaling is only possible via receptor binding. The antibody V2 binds to human RANTES and Met-RANTES, but not to the rat equivalent of CCL5. Thus, the antibody mainly stained Met-RANTES bound to GAGs or to CCR1 or CCR5.

### 2.2. Infiltration of Inflammatory Cells via the Choroid and the RPE (Outer Blood–Retina Barrier)

**Figure 2:**
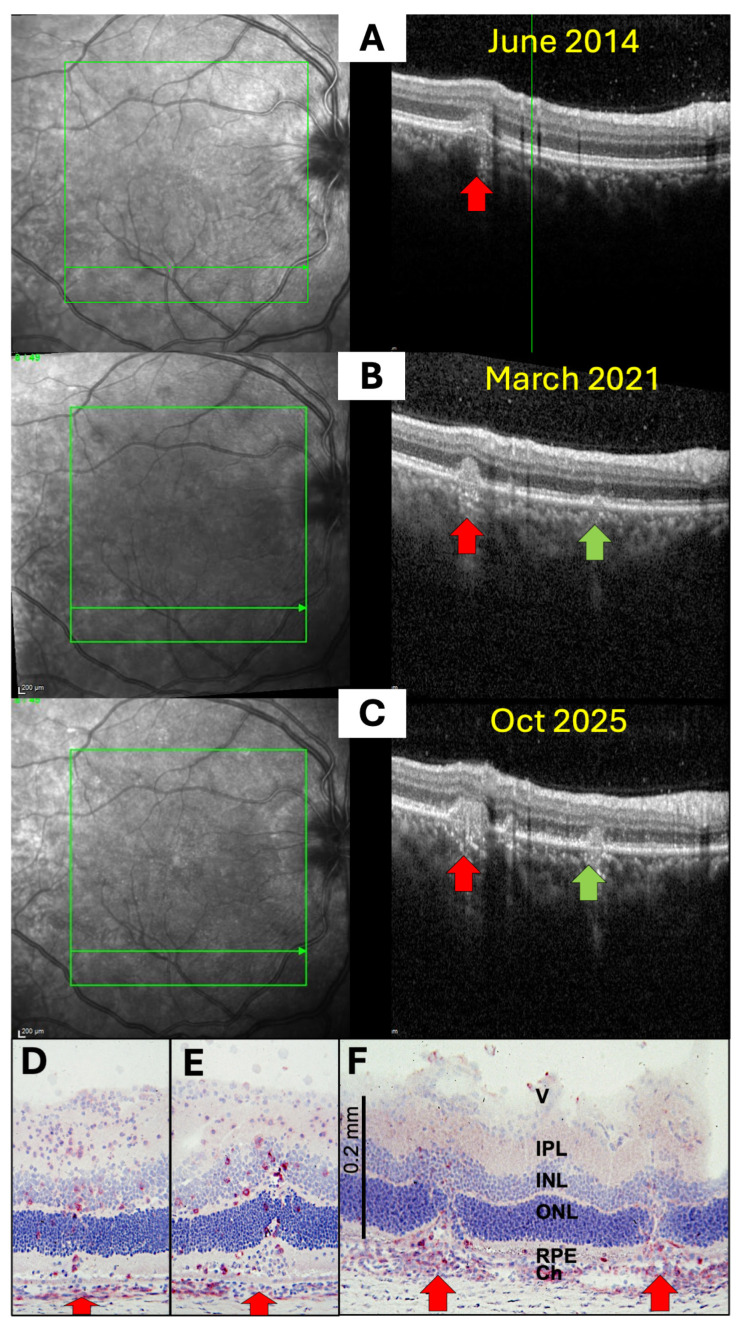
SD-OCT images of a 34-year-old female patient with multifocal choroiditis and progressing lesions over 11 years (**A**–**C**). Infiltration from the choroid with destruction of the RPE and the outer nuclear layer (right panel: red and green arrows mark identical lesions detectable over the 11 years of observation). The left panel shows the confocal scanning laser ophthalmoscope (cSLO) images, with the OCT scan location indicated by a green arrow within the green rectangle (screen range). The vertical green line in (**A**) (right panel) indicates the position of the cursor at the time of capturing the image. The scan location was the same in all three images. Similar pictures were obtained from PDSAg-induced rat EAU (**D**–**F**), which show red staining of CD4^+^ T helper cells and macrophages infiltrating the retina via the RPE, resulting in focal destruction of the superincumbent layers of the retina and the RPE (**E**,**F**). The RPE is still intact in the early stages in both human uveitis and rat EAU (**A**,**D**,**E**), but focal destruction of the RPE layer increases with progressive disease (**B**,**C**,**F**). V: vitreous, IPL: inner plexiform layer, INL: inner nuclear layer, ONL: outer nuclear layer, RPE: retinal pigment epithelium, Ch: choroid.

Several uveitis entities originate in the choroid, and inflammatory infiltrates spread from there to the retina via the RPE. Figure 2A–C show OCT scans of a patient with multifocal choroiditis, which is characterized by multifocal inflammation of the superficial choriocapillaris with yellow-orange, round or oval, sometimes elevated lesions, typically >250 µm in size and resulting in punched-out atrophic scars observed by fundoscopy. Previous studies have described the stages that acute lesions in multifocal choroiditis undergo on OCT [16]: First, small nodular lesions appear at the level of the RPE, causing an upward bulge that resembles a small pigment epithelium detachment. Then, hyperreflective infiltrates expand through the RPE into the outer retina, leading to the disorganization of the retinal layer architecture of varying severity. With successful treatment, these disorganizations and the hyperreflective infiltrates of the retina disappear. However, a certain degree of RPE and outer retinal atrophy remains, leaving behind the punched-out focal atrophic lesions that are visible by fundoscopy.

Histologies of rat EAU eyes demonstrate that immune cell infiltration is focal and not evenly distributed across the choroid–RPE interface (Figure 2D–F). An accumulation of CD4^+^ T helper cells and monocytes/macrophages occurs in the choroid just below the infiltration site. As seen in Figure 2E, only a few CD4^+^ cells invade the photoreceptor layer; the other, unstained cells may be CD8^+^ T cells, B cells, natural killer (NK) cells, monocytes/macrophages or granulocytes. Interestingly, despite the massive infiltration, the RPE monolayer remains intact for an extended period (Figure 2D,E) but is ultimately destroyed by continuing inflammation. Similar findings can be seen in the OCT scans, which showed hyperreflectivity in the choroid under the lesions (Figure 2B,C). Furthermore, an increasing number of infiltrating cells can be found within the photoreceptor and the nuclear layers (Figure 2D–F), which indicates that inflammation spreads beyond the obvious foci. These small, hyperreflective irregularities, which are also referred to as hyperreflective foci, may be represented in the OCT scans [17] (Figure 2B,C, right panels).

**Figure 3:**
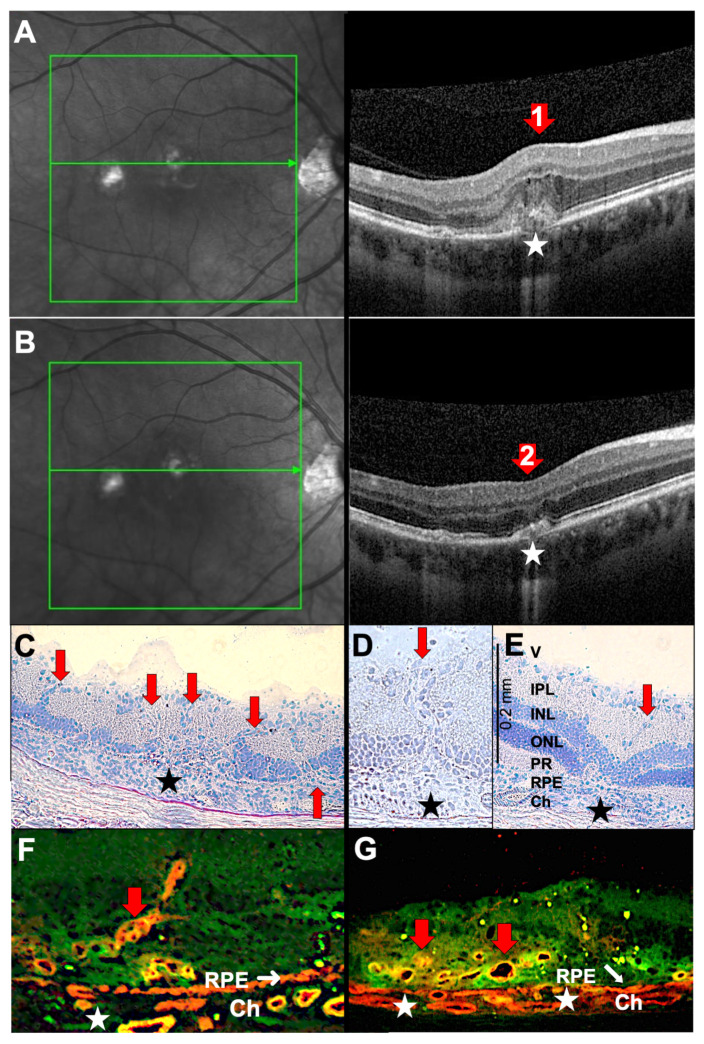
Choroidal neovascularizations (CNVs) in uveitis of a 31-year-old male patient with PIC. (**A**,**B**) SD-OCT B-scans (**right**) and corresponding cSLO images (**left**). The left panel shows the confocal scanning laser ophthalmoscope (cSLO) images, with the OCT scan location indicated by a green arrow within the green rectangle (screen range). (**A**) Active CNV with thickening of the retina, development of cystoid macular edema and hyporeflective spaces, indicating fluid accumulation in the Henle fiber layer and inner nuclear layer, as well as subretinal hyperreflective lesions with RPE disruption (red arrow 1). (**B**) The same scan region 4 weeks after one intraretinal injection of ranibizumab (red arrow 2). (**C**,**D**) Rat retina histology (cryosection, hematoxylin stained) of PDSAg-induced EAU with severe retinal destruction extending to the inner nuclear layer and new vessels sprouting from the choroid to the ganglion cell layer (red arrows). (**E**) The same EAU induction as in C and D, but the rat was treated with systemic cyclosporine A from onset of uveitis until the end of the experiment at day 39. Cyclosporine A suppressed T cells, and thus, their VEGF production, which led to suppression of CNV. Black asterisks (**C**–**E**) mark focal RPE destruction and underlying choroidal infiltration. V: vitreous, IPL: inner plexiform layer, INL: inner nuclear layer, ONL: outer nuclear layer, PR: photoreceptor, RPE: retinal pigment epithelium, Ch: choroid. (**F**,**G**): Rat retina histologies of PDSAg-induced EAU with CNV and immunofluorescence staining for CD31/PECAM (red) and CD146/MCAM (green). The overlay of both stainings is shown in orange or yellow, respectively. Red arrows mark CNVs. (**F**) Longitudinal sections; (**G**) cross-sections through the new blood vessels. White asterisks mark focal RPE destruction, white arrows indicate the RPE. Ch: choroid.

Choroidal lesions are sometimes followed by the uncontrolled sprouting of new vessels from the choriocapillaris into the retina. This results in focal destruction of the RPE layer, as seen in punctate inner choroidopathy (PIC, a distinct type of multifocal choroiditis with similar, but smaller, lesions ≤ 150 µm in diameter; it typically occurs in young adult myopic women [18]) (Figure 3). Previously, it was believed that VEGF originated from stressed RPE cells or choroidal macrophages. However, we demonstrated that T cells can secrete VEGF, inducing CNVs (choroidal neovascularizations) [6]. In the rat model, PDSAg-specific, but not R14-specific, T cells produce VEGF, and thus, cause growth of new vessels (Figure 3C–E), which can be observed after the peak of inflammation. The CNVs increase in number and intensity even after clinically visible inflammation resolves, because some VEGF-secreting, autoreactive T cells remain within the retina for several weeks in rat EAU [7]. CNV formation can be prevented or alleviated by suppressing the T cells in EAU (Figure 3D and [6]). In the patient shown here, regression of CNVs was induced by intraocular injection of the anti-VEGF antibody ranibizumab (Figure 3B).

**Figure 4:**
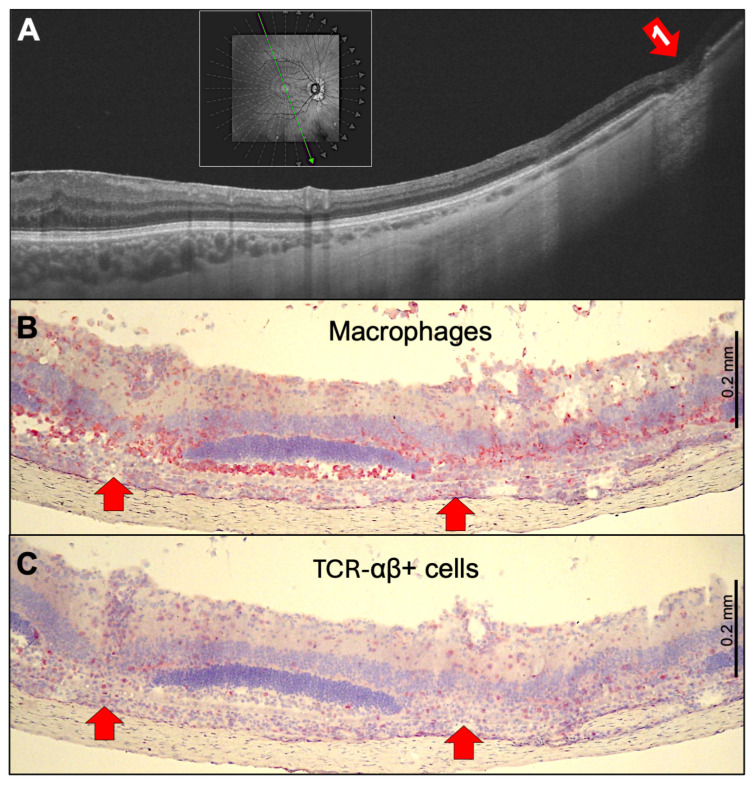
(**A**) Ultra-widefield-swept-source OCT B-scan of the right eye of a 52-year-old female patient with multifocal choroiditis showing peripheral atrophy of the choroid, the RPE, and the outer retinal layers (red arrow 1). The green arrow in the rectangular insert indicates the orientation of the OCT section, the gray arrows indicate other potential sections. (**B**,**C**) In acute inflammation, PDSAg-induced rat EAU histologies show massive infiltration of inflammatory ED1^+^ macrophages (**B**) and fewer TCR-αβ^+^ T cells (**C**), shown by the red staining. There is also severe destruction of the outer retinal layers, and some focal lesions of the RPE (red arrows). These can be detected in human OCT as residual outer retina and RPE loss ((**A**), red arrow), while active inflammation subsided. In rat EAU, perivascular inflammation of retinal vessels in the ganglion cell layer can be observed in addition. (**B**,**C**) R14-immunized, serial sections of the same rat eye.

In addition to the small, focal lesions shown in Figure 2, choroiditis can result in broader atrophic lesions affecting multiple layers of the retina (Figure 4). These lesions destroy the RPE and, consequently, the outer blood–retina barrier. The OCT scan (Figure 4A) showed that the peripheral lesion extended to the inner nuclear layer. This is also evident in PDSAg-induced rat EAU in Figure 4B,C, where infiltrating macrophages (ED1 staining, Figure 4B) and T cells (αβ T cell receptor staining, Figure 4C) can be visualized separately. This demonstrates the relationship between a small number of immigrating T cells and many infiltrating macrophages. Interestingly, despite the complete destruction of the photoreceptor’s outer segments, the RPE layer remained mostly intact.

### 2.3. Infiltration via Retinal Vessels (Inner Blood–Retina Barrier)

Similar to rat EAU, in which retinal vasculitis and infiltration via the choroid and RPE can be observed in PDSAg-induced EAU, there are also patients with chorioretinitis, showing inflammation of both the choroid and retinal vessels.

**Figure 5:**
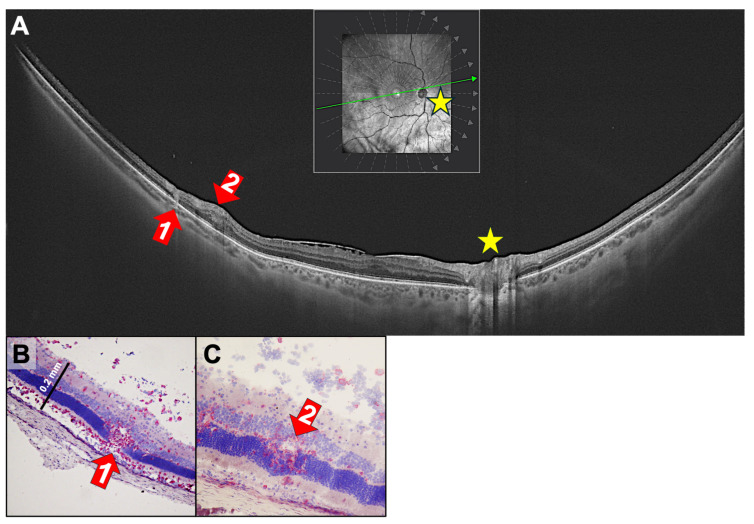
(**A**) Radial ultra-widefield-SS-OCT B scan of the right eye of a 69-year-old male patient with chronic intermediate uveitis and peripheral vasculitis. Perivascular hyperreflective infiltrates with thickening and partial disruption of the inner retinal layers are marked by red arrows 1 and 2. A hyperreflective transretinal infiltrate with discontinuation of the outer retinal layers is seen above red arrow 1. Yellow asterisks mark the optic nerve head. The green arrow in the rectangular insert indicates the orientation of the OCT section, the gray arrows indicate other potential sections. (**B**,**C**) Similar features can be found in rat EAU retinas after PDSAg immunization with focal destruction of the outer and inner nuclear layers (red arrows correspond to the respective lesions in (**A**)). Histologies reveal destruction of photoreceptor outer segments and outer retinal layers with infiltration of CD4^+^ T cells and macrophages (red staining).

One patient with intermediate uveitis (Figure 5A) had two retinal lesions that originated from retinal vessels and resulted in extended destruction of retinal layers. The focus of inflammation in intermediate uveitis is the peripheral retina, the posterior ciliary body, and superficial retinal vessels (typically veins) as well as small capillaries throughout the retina [19]. There is a statistical association with multiple sclerosis. This patient was under immunosuppressive therapy with cyclosporine and presented with clinically inactive disease at the day of image acquisition. However, his OCT scans showed perivascular hyperreflective infiltrates with thickening and partial disruption of the inner retinal layers and a hyperreflective transretinal infiltrate with discontinuation of the outer retinal layers. These findings indicate residual inflammatory activity (Figure 5A). Similar retinal destructions can be seen in the rat sections in Figure 5B,C. Lesion No. 2 in the OCT scan (Figure 5A) appeared to spread from retinal capillaries in the outer plexiform layer, as seen in the rat histologies of Figure 5B,C.

Birdshot chorioretinopathy is defined as bilateral multifocal choroiditis with cream-colored or yellow-orange oval or round spots deep in the stroma of the choroid; focal spots of retinitis, which are frequently above the choroidal infiltrates; and retinal vasculitis. There is a strong association between birdshot chorioretinopathy and HLA-A29 [20]. We used this disease as an example of infiltration via retinal vessels alongside simultaneous choroidal inflammation.

**Figure 6:**
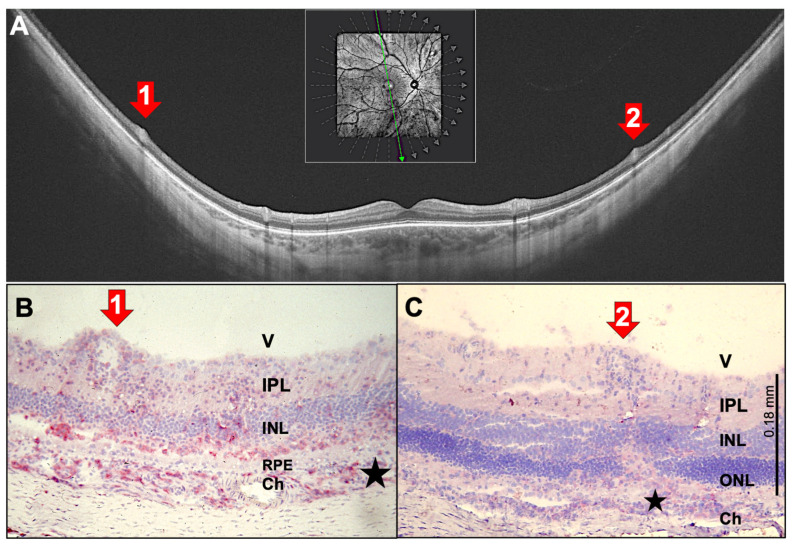
(**A**) Ultra-widefield-SS-OCT B scan of a 47-year-old female patient with birdshot chorioretinopathy (BCR) and myopia with vasculitis and perivascular swelling. Red arrows 1 and 2 indicate the location of thickened peripheral venules. The green arrow in the rectangular insert indicates the orientation of the OCT section, the gray arrows other potential sections not shown in this figure. (**B**,**C**) Rat eyes with EAU; perivascular inflammation of retinal vessels (red arrows) and an inflammatory cell infiltrate ((**B**): red staining of CD4^+^ cells) are visible, as well as the total destruction of photoreceptors (outer segments and outer nuclear layer). There are also choroidal swelling and focal destruction of the RPE (black asterisks). V: vitreous, IPL: inner plexiform layer, INL: inner nuclear layer, ONL: outer nuclear layer, RPE: retinal pigment epithelium, Ch: choroid. (**B**) R14-immunized; (**C**) PDSAg-immunized.

Despite receiving treatment with a combination of adalimumab and mycophenolate mofetil, a patient with birdshot chorioretinopathy exhibited peripheral vasculitis accompanied by additional perivascular swelling (Figure 6A). The thickened venules corresponded to mild exudation on fluorescein angiography and were graded as mild vasculitis clinically. Corresponding rat histologies are shown in Figure 6B,C. In the vasculitic areas, the outer retinal layers appear thinned and granular. The macula was unaffected (Figure 6A).

### 2.4. Inflammation of the Inner Retinal Vessels and Infiltration of the Vitreous

Most patients with intermediate uveitis suffer from vasculitis of the retinal vessels and cellular infiltration of the vitreous. These conditions lead to floaters and an epiretinal accumulation of inflammatory cells that appear as “snowballs” and “snowbanks” [21].

**Figure 7:**
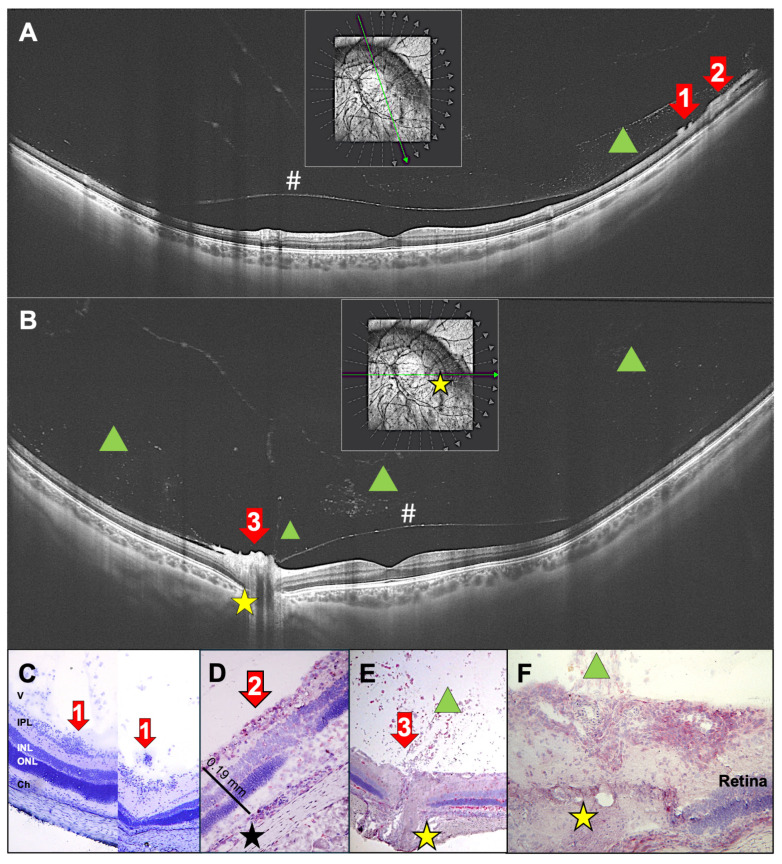
(**A**) Ultra-widefield-SS-OCT scans of a 31-year-old female patient with newly diagnosed, untreated intermediate uveitis, showing inferior snowballs and snowbanks (red arrows 1,2). (**B**): The same patient (same eye, different OCT section) is shown with papillitis (red arrow 3). Yellow asterisks mark the optic nerve head, and green triangles indicate cells infiltrating the vitreous. The posterior hyaloid is detached (#). (**A**,**B**): The green arrow in the rectangular insert indicates the orientation of the OCT section, the gray arrows indicate other potential sections. (**C**) Rat histologies showing snowballs (red arrows 1) at the vitreous membrane. (**D**) A rat retina with late-stage EAU showing a tight accumulation of inflammatory cells in the ganglion cell layer (snowbank, red arrow 2) and focal retinal destruction. The black asterisk marks focal RPE destruction. (**E**,**F**) Papillitis (red arrow 3) with CD4^+^ cells infiltrating the vitreous from the papilla (green triangle). Photoreceptors are destroyed and infiltrated by inflammatory cells (red staining). (**F**) A magnification of the optic nerve head (yellow asterisk) shows inflamed central retinal veins and cellular exudates. (**C**) Hematoxylin staining only; (**D**–**F**) CD4^+^ cell staining in red. V: vitreous, IPL: inner plexiform layer, INL: inner nuclear layer, ONL: outer nuclear layer, Ch: choroid. (**C**,**D**) PDSAg-immunized; (**E**,**F**) IRBP-immunized.

One patient with intermediate uveitis presented with peripheral snowballs and snowbanks, which are aggregates of inflammatory cells in the vitreous and the posterior hyaloid membrane. The patient also presented with vitreous infiltrates and papillitis (Figure 7). Histologies from EAU rat eyes showed dense clusters of inflammatory cells within or next to the ganglion cell layer (Figure 7C,D). These accumulations were either round (snowballs) or elongated (snowbanks) and represented the hyperreflective structures seen in OCT scans (Figure 7A,C,D). Cellular infiltration of the vitreous at the optic nerve, as seen in the OCT scan (Figure 7B), seems to originate from the inflamed optic nerve head, as also seen in the rat histologies (Figure 7E,F).

**Figure 8:**
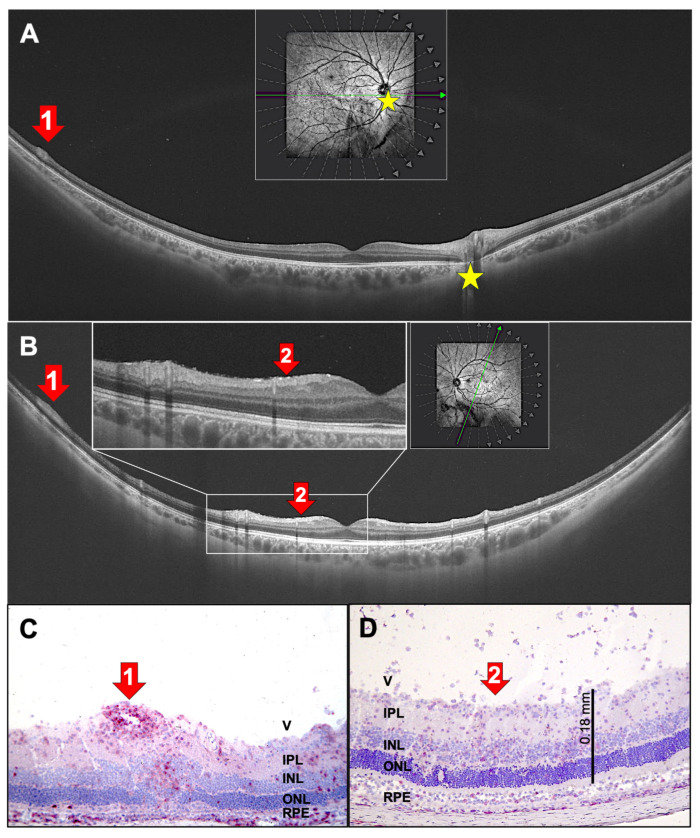
(**A**,**B**) The right (**A**) and left (**B**) eyes of a 31-year-old patient with untreated intermediate uveitis with vasculitis (red arrows 1, yellow asterisk marks the papilla). The green arrow in the rectangular insert indicates the orientation of the OCT section, the gray arrows indicate other potential sections. (**C**,**D**) Rat EAU histologies with red staining of CD4^+^ cells. (**A**–**C**) Red arrows 1 indicate peripheral perivascular swelling with vasculitis. (**B**,**D**) Red arrows 2 indicate inflammatory cells on the retinal surface (**B**,**D**). V: vitreous, IPL: inner plexiform layer, INL: inner nuclear layer, ONL: outer nuclear layer, RPE: retinal pigment epithelium. (**C**) IRBP-immunized; (**D**) PDSAg-immunized.

Figure 8A,B show typical intermediate uveitis findings, including peripheral vasculitis. The corresponding rat histology is shown in Figure 8C. Additionally, the OCT scan of the left eye showed very small, white, hyperreflective spots on the epiretinal surface and within the nerve fiber layer (Figure 8B). A careful investigation revealed only sparse infiltration of inflammatory cells in the vitreous. In a rat EAU eye, inflammatory cells are distributed throughout all layers of the retina, as well as in the nerve fiber and ganglion cell layers (Figure 8D). Some cells also infiltrate the vitreous.

## 3. Discussion

Human uveitis encompasses a wide range of clinical presentations and is classified as infectious or non-infections and anterior, intermediate, or posterior uveitis with granulomatous, nodular, or non-granulomatous/fibrinous inflammation. Very little is known about the immune pathomechanisms or the autoantigens of the immune response. However, some retinal antigens have been identified that are recognized by peripheral lymphocytes of uveitis patients. These include the retinal S-antigen, IRBP [8], cRALBP [9], and tyrosinase family proteins for Vogt–Koyanagi–Harada disease (VKHD) [22]. VKHD is characterized by sterile meningitis, vitiligo, and chronic panuveitis with serous retinal detachment [23]. Moreover, certain HLA molecules and polymorphisms of a few other molecules have been identified as being more or less strongly associated with specific types of uveitis. Finally, the microbiome has been implicated [24].

Similarly, clinically classified presentations of uveitis may manifest as different conditions. Noninfectious uveitis is subgrouped according to the clinical presentation, as defined by a slit lamp examination, fundoscopy, angiography of the retinal and choroidal vessels, and OCT, which provide insight into the retinal structure. OCT has led to a much better understanding of many eye diseases, including uveitis. However, thus far, the resolution of the scans only allows for an estimation of the inflammatory process in the retina, not a precise determination. In this manuscript, we analyzed immune cell migration and infiltration into neuroretinal tissue in an EAU rat model. Our findings reveal significant parallels with OCT imaging in human patients with uveitis.

Although animal models of uveitis have their limitations, they have helped us to better understand the immunological pathomechanisms of the disease and develop therapies. EAU is a model of panuveitis with high inflammatory activity that leads to severe tissue damage within days. This contrasts with most cases of human uveitis, which have slower and less severe inflammation. Nevertheless, if no treatments were administered, the final tissue damage would be similar. Typical patterns of inflammatory activity include perivascular infiltrates (vasculitis), immune cell accumulation at the vitreoretinal interface or posterior hyaloid space (snowballs or snowbanks), and infiltration of the outer retinal layers by immune cells that have migrated from the choroid.

Experimental uveitis is typically induced in certain strains of mice and rats. Despite being genetically identical and kept under the same environmental conditions, these experimental animals exhibit variations in disease progression. Lewis rats reveal individual differences in the clinical manifestations and histological findings of their uveitis.

The two major autoantigens for experimental autoimmune uveitis (EAU) in Lewis rats are the retinal S-antigen (S-Ag) and IRBP [3]. Rat EAU is defined as pan-uveitis; however, IRBP-induced EAU usually affects both the anterior and posterior segments, whereas S-Ag-induced uveitis primarily affects the posterior segment. The extracellular molecule IRBP transports vitamin A derivatives between photoreceptors and the RPE. S-Ag is an intracellular photoreceptor protein and is taken up by RPE cells when the outer PR segments are phagocytosed. We developed two uveitis models in Lewis rats, using two different antigen peptides for immunization: PDSAg, which is derived from retinal S-Ag, and R14, which is derived from IRBP. These antigens induce two distinct types of uveitis with respect to their disease course and pathological findings.

PDSAg-induced uveitis has only one visible clinical course of inflammation that resolves approximately three weeks after immunization. Around 28 days after immunization, new vessels grow from the choroid throughout the retina. We found that PDSAg-specific T helper cells secrete VEGF, inducing CNV. Suppressing T cells and their VEGF secretion inhibits CNV formation [6].

These results suggest that CNV formation in uveitis is primarily caused by T cell VEGF secretion rather than by RPE. Since we also demonstrated VEGF secretion with human T cells, we propose that the sprouting of new blood vessels in the eyes of uveitis patients may also be caused by autoreactive T cells rather than by VEGF produced by the RPE or macrophages [6]. This notion is supported by the observation that choroidal neovascularization in human uveitis exhibits different characteristics than neovascularization resulting from other diseases, such as age-related macular degeneration (AMD), venous occlusion, or diabetes. For this reason, it has been named inflammatory choroidal neovascularization (iCNV) [25,26]. As in the animal model, immunosuppression reduces iCNV recurrence in humans, further supporting the idea that VEGF-dependent neovascularization is driven by T cells [27].

As previously mentioned, the autoantigen specificity of the immune response in patients with uveitis is unknown. However, certain types of uveitis may exhibit a strong preference for one or a few autoantigens and their epitopes, which is similar to the situation in our rat model.

These different autoantigens may explain the differences in retinal damage observed after immunization with antigens such as the S-antigen and IRBP. IRBP-induced uveitis tends to affect the anterior part of the eye more, possibly because IRBP mRNA has been found in the ciliary body as well [28]. In the posterior part of the eye, IRBP is present in the vitreous and the interphotoreceptor matrix, which is located between the retinal pigment epithelium (RPE) and the external limiting membrane. There, IRBP is secreted by photoreceptor rods and cones. Under physiological conditions, IRBP is taken up by RPE cells, where it is recycled, but not degraded. This process potentially excludes RPE cells from presenting IRBP peptides and may explain why, in IRBP/R14-induced EAU, infiltrating macrophages can completely destroy the outer segments of photoreceptors while leaving the RPE intact.

Because IRBP floats freely in the interphotoreceptor matrix and elsewhere in the eye, any cell that qualifies as an APC (such as retinal microglia) can easily take it up [29]. Microglia processes extending to the outer plexiform layer and presenting IRBP peptides can come into contact with retinal capillaries. This may attract IRBP-specific T cells, leading to increased infiltration between the two nuclear layers, as can be observed in IRBP/R14-induced disease [30].

The situation is different with the S-antigen. Upon phagocytosis of outer PR segments, S-Ag also appears in the retinal pigment epithelium (RPE), where it is degraded. Along with several growth factors, S-Ag fragments can travel to the choriocapillaris [31], where they are taken up and presented by local macrophages. These macrophages can then attract lymphocytes from the vasculature to the RPE and into the retina. This attracts inflammatory cells, which facilitates RPE destruction. RPE cells can present antigens as well, but they usually express HLA class II molecules only under inflammatory conditions. Destruction of the RPE, which forms the outer blood–retina barrier, allows inflammatory cells to invade and severely damage the retinal architecture. In humans, RPE destruction followed by scar formation leads to “white dots”, which are often observed during fundoscopic examinations in chorioretinopathies.

Treating rats with Met-RANTES three to six days after adoptive transfer abrogated only PDSAg-induced EAU, but not R14-induced EAU [15]. Met-RANTES, a chemokine receptor antagonist, competitively inhibits the binding of CCL2/RANTES to CCR1 and CCR5, but does not induce signaling. In EAU, activated autoreactive T cells enter the eye approximately three days before the onset of clinical disease, i.e., infiltration of inflammatory cells. Once they recognize their respective antigen, they become reactivated and can recruit inflammatory cells to the inner eye a few days later [12]. Since PDSAg- and R14-specific T cells do not differ in their expression of respective chemokine receptors, we propose that Met-RANTES primarily acts on inflammatory cell infiltration rather than on T cells. In PDSAg-induced EAU, this infiltration occurs preferentially from the choroid via the RPE and may be blocked by Met-RANTES a few days before the expected onset of clinical disease (data available under series record GSE19652 on the NCBI Gene Expression Omnibus webpage: http://www.ncbi.nlm.nih.gov/geo/) [4].

Like many animal models, one limitation of this study is the difficulty of applying the results to human disease. The two antigens and the two types of uveitis that they induce are just two examples from the EAU rat model. They demonstrate the differences in immune responses to specific antigens or epitopes and their effects on target tissues. The same may occur in patients, likely through a combination of immune responses rather than a single epitope specificity. Other autoantigens may result in different T cell types with additional characteristics, inducing different clinical manifestations and effects on ocular tissues than those shown here. Regardless of the autoantigen, inflammation patterns seem to repeat and resemble each other. This is evident in the close correspondence between human OCT images and rat histology images.

The strength of this analysis lies in its detailed, tissue-level perspective on inflammation. There is an unmet need for a deeper understanding of the immunological basis of imaging signs in human uveitis. Currently, multimodal imaging is primarily used to diagnose uveitis and, to a lesser extent, to monitor therapeutic success. Identifying imaging criteria that indicate activity could make controlling uveitis activity far more precise. Thus far, OCT has primarily been used at the posterior pole, e.g., to assess the presence of cystoid macular edema (CME). Widefield imaging allows for the inclusion of peripheral signs of active disease in routine assessments and enables the monitoring of the efficacy of immunomodulatory therapy. Ideally, relapses in uveitic inflammation would be reliably detected before visible clinical signs appear on the slit lamp and before harmful complications occur. Reliable imaging parameters for uveitis activity could serve as endpoints to evaluate the efficacy of uveitis treatments. These endpoints could solve the problem of irregular and unpredictable endpoints that lead to failed phase 3 trials [32]. The detailed insights into rat histologies presented here could guide future basic science and clinical imaging studies.

## 4. Materials and Methods

### 4.1. Patient Recruitment and Characteristics

Eight patients with various types of uveitis from our outpatient department underwent diagnostic OCT imaging of the retina. Basic clinical data were collected from each patient, and brief case descriptions can be found in the figure legends. Ethical approval and informed consent were obtained for all patient data (see the “Institutional Review Board Statement” section below).

### 4.2. OCT Imaging

Spectral-domain OCT imaging was performed on a Heidelberg Spectralis device (Heidelberg Engineering, Heidelberg, Germany). Macular volume scans were recorded with the following settings: 20° × 20° (5.9 × 5.9 mm), 49 horizontal B-scans, 18 ≤ ART ≤ 30. Swept-source ultra-widefield imaging was performed using an Intalight^®^ DREAM OCT™ (SVision Imaging Ltd., Luoyang, China). Images were acquired using a star-shaped scan pattern of 18 radial B-scans centered on the fovea (scan length of 26 mm, depth of 6.3 mm).

### 4.3. Histological Case Selection Process and Analysis

We re-examined our existing research database of routinely prepared histological images for EAU scoring from previous experiments. The analysis focused on cell types, inflammation sites, inflammatory patterns, and clinical transferability in terms of their correspondence to human OCT findings. These histological images have not been published previously. All experiments were approved by the Government of Upper Bavaria and were performed in accordance with the German and European laws, as well as the ARVO (Association for Research in Vision and Ophthalmology) recommendations for the use of animals in vision research. The approval codes and dates are provided at the end of the manuscript.

### 4.4. Induction of Experimental Uveitis

Male and female albino Lewis rats were either obtained from the Central Institute for Laboratory Animal Breeding (Hannover, Germany), RCC (Basel, Switzerland), or Janvier (Le Genest St. Isle, France). The rats were also bred in our own colony and maintained under specific pathogen-free conditions with water and food ad libitum and used for experiments at the age of 6–8 weeks.

The rats were immunized subcutaneously in both contralateral hind legs with a total of 15 μg of retinal S-Ag peptide PDSAg (amino acids 342–355, FLGELTSSEVATEV (1)), or 15 μg of IRBP-peptide R14 (amino acids 1169–1191, PTARSVGAADGSSWEGVGVVPDV). The peptides were emulsified in an equal volume of complete Freund’s adjuvant (CFA) containing 2.5 mg/mL of Mycobacterium tuberculosis strain H37Ra (DIFCO, Hedinger, Stuttgart, Germany) and phosphate-buffered saline. IRBP was prepared from bovine eyes as described in (3), and its purity was verified by gel electrophoresis. The peptides PDSAg and R14 were purchased from Biotrend, Cologne, Germany. No B. pertussis or pertussis toxin were used as an additional adjuvant. A total volume of 200 µL of the emulsion was injected subcutaneously into each hind leg.

### 4.5. Immunohistochemistry

The eyes were collected for a histological analysis after the termination of the experiments between days 21 and 24 post-immunization. The enucleated eyes were embedded in Tissue Tec OCT compound (Sakura Finetek, Torrance, CA, USA) and snap-frozen in methylbutane at −70 °C. Frozen sections (8 μm) were placed on slides coated with poly-L-lysine (SIGMA, Deisenhofen, Germany), fixed in ice-cold acetone, and air-dried. Next, mouse anti-rat monoclonal antibodies specific for CD4 (W3/25, provided by Th. Hünig, Würzburg, Germany), TCR-αβ (R73, provided by Th. Hünig, Würzburg, Germany), or ED1 (monocytes/macrophages, Biozol, Eching, Germany) were added for one hour. 

To demonstrate Met-RANTES binding to cells in the uveitic retina, we performed sandwich staining by adding Met-RANTES, which binds to glycosaminoglycans on cell surfaces as well as to the receptors CCR1 and CCR5. Then, we added the antibody VL2, which is specific for Met-RANTES and human CCL5, and was a gift from Peter J. Nelson. We added Met-RANTES or PBS (the control) to the sections for 1 h at RT, after which we washed the sections with Tris/HCl (pH of 7.5) buffer. Then, we added mouse anti-human RANTES antibody VL2 for another hour before continuing the staining process.

After washing the slides once more with Tris buffer, they were incubated with a (Fab’)_2_ rat anti-mouse Ig antibody (linking primary antibodies with the APAAP complex) for 30 min. Finally, we added the alkaline phosphatase–anti-alkaline phosphatase (APAAP) complex (1:80, Dianova, Hamburg, Germany). We used new fuchsin as the substrate, as described in (4). In some cases, we omitted the immunohistochemical staining step and counterstained the slides with hematoxylin only. The slides were mounted with glycerol gelatin and imaged with a Zeiss Axioskop 2plus (Carl Zeiss, Oberkochen, Germany). Photographs were taken with a Sony CyberShot DSC-S70 3.3 MP digital camera (Sony Deutschland GmbH, Cologne, Germany).

## Data Availability

The original contributions presented in this study are included in the article. Further inquiries can be directed to the corresponding authors.

## Abbreviations

The following abbreviations are used in this manuscript:

CME	cystoid macular edema
CNV	choroidal neovascularization
cSLO	confocal scanning laser ophthalmoscope
EAU	experimental autoimmune uveitis
IRBP	interphotoreceptor retinoid-binding protein
OCT	optical coherence tomography
PDSAg	peptide derived from retinal S-antigen
PIC	punctate inner choroidopathy
RPE	retinal pigment epithelium
R14	peptide derived from IRBP
S-Ag	S-antigen, retinal soluble antigen
SD-OCT	spectral-domain optical coherence tomography
SS-OCT	swept-source optical coherence tomography
VEGF	vascular endothelial growth factor
